# Enhanced Thermal Conductivity of Polymer Composite by Adding Fishbone-like Silicon Carbide

**DOI:** 10.3390/nano11112891

**Published:** 2021-10-28

**Authors:** Juncheng Xia, Yue Qin, Xianzhe Wei, Linhong Li, Maohua Li, Xiangdong Kong, Shaoyang Xiong, Tao Cai, Wen Dai, Cheng-Te Lin, Nan Jiang, Shuangquan Fang, Jian Yi, Jinhong Yu

**Affiliations:** 1 School of Mechanical Engineering, Yangzhou University, Yangzhou 225009, China; xiajuncheng@nimte.ac.cn; 2 Ningbo Institute of Materials Technology and Engineering, Chinese Academy of Sciences, Ningbo 315201, China; qinyue@nimte.ac.cn (Y.Q.); weixianzhe@nimte.ac.cn (X.W.); lilinhong@nimte.ac.cn (L.L.); limaohua@nimte.ac.cn (M.L.); kongxiangdong@nimte.ac.cn (X.K.); xiongshaoyang@nimte.ac.cn (S.X.); caitao@nimte.ac.cn (T.C.); daiwen@nimte.ac.cn (W.D.); linzhengde@nimte.ac.cn (C.-T.L.); jiangnan@nimte.ac.cn (N.J.); 3 Center of Materials Science and Optoelectronics Engineering, University of Chinese Academy of Sciences, Beijing 100049, China

**Keywords:** silicon carbide, polyvinylidene fluoride, thermal conductivity, electronic packaging

## Abstract

The rapid development of chip technology has all put forward higher requirements for highly thermally conductive materials. In this work, a new type of material of Fishbone-like silicon carbide (SiC) material was used as the filler in a polyvinylidene fluoride (PVDF) matrix. The silicon carbide/polyvinylidene fluoride (SiC/PVDF) composites were successfully prepared with different loading by a simple mixing method. The thermal conductivity of SiC/PVDF composite reached 0.92 W m^−1^ K^−1^, which is 470% higher than that of pure polymer. The results show that using the filler with a new structure to construct thermal conductivity networks is an effective way to improve the thermal conductivity of PVDF. This work provides a new idea for the further application in the field of electronic packaging.

## 1. Introduction

With the continuous advancement of chip technology, high frequency and power gradually become the developing direction of integrated electronic devices. This trend causes more waste heat, which is generated from its limited volume. If the waste heat cannot be removed in time, the routine operation of a device with continuous high working temperature will be affected and probably even be damaged. Therefore, the heat dissipation problem of chip devices continues to receive more attention [1,2,3,4,5,6,7,8]. Polymers with the advantages of chemical resistance, high-temperature resistance, oxidation resistance, and weathering resistance are widely used as thermal interface materials (TIM) [9,10,11,12,13,14]. However, most polymers exhibit typically poor thermal conductivity, such as the thermal conductivity of polyvinylidene fluoride (PVDF), polydimethylsiloxane (PDMS), polystyrene (PS), etc., ranging from 0.1 to 0.4 W m^−1^ K^−1^ [15], which significantly limits their applications as TIMs. In order to solve this problem, functional fillers of different shapes and types with high thermal conductivity are added into polymers to improve the heat transportation efficiency [16,17,18,19,20,21,22,23]. Wang et al. enhanced the thermal conductivity of carbon fiber/polymer composites through SiC nanowires/graphene hybrid nanofillers [24]. Spinelli et al. explored the thermal conduction research of polylactic acid (PLA) filled with two types of carbon nanotubes and two types of graphene nanosheets or their appropriate combination [25].

Polyvinylidene fluoride (PVDF) is a thermoplastic polymer with comprehensive properties, such as excellent mechanical strength, thermal stability, chemical stability, and easy processing [26]. However, due to the low glass transition temperature of PVDF (*T*_g_ = −35 °C), its thermal performance is not so high [27], which requires the use of inorganic fillers with excellent thermal properties to reinforce PVDF. Silicon carbide (SiC) with high thermal conductivity and excellent mechanical properties is considered to be an ideal filler for polymer composites [28,29,30]. Shen et al. prepared epoxy/silicon carbide nanowires (SiCNWs) nanocomposites by mechanical blending. The thermal conductivity of the polymer composites with 3.0 wt% filler content reached 0.45 W m^−1^ K^−1^, which is approximately a 106% enhancement compared with pure epoxy [31]. Yao et al. designed a vertically interconnected honeycomb SiCNWs network prepared in epoxy resin by freezing casting and thermal sintering. Compared with the pure matrix, the composites reach a higher through-plane thermal conductivity (1.67 W m^−1^ K^−1^), which is equivalent to a significant increase of 406.6% per 1 vol% loading [32]. Wang et al. used aminated SiC nanowires to prepare f-SiC nanowire/PVDF composites with 13.8 vol% filler content by hot-pressing after casting on glass. The obtained composite material has superior dielectric properties, and its thermal conductivity is twice that of pure PVDF [33]. In addition, Wang et al. also used hexagonal silicon boron (hBN) nanosheets and functionalized silicon carbide (f-SiC) nanowires to prepare PVDF dielectric composites in the same way, and its thermal conductivity reaches 1.41 W m^−1^ K^−1^ [34]. In this work, Fishbone-like SiC fillers were added into PVDF to prepare a polymer composite by blending and hot-pressing.

## 2. Experimental Section

### 2.1. Materials

SiC powders (CVD grade, 100–600 nm in diameter) were provided by Changsha Sinet Advanced Material Co., Ltd. (Changsha, China). N, N-Dimethylformamide (DMF, >99.0 wt%) solution and polyvinylidene fluoride (PVDF) were provided by Sinopharm Chemical Reagent Co., Ltd. (Shanghai, China).

### 2.2. Preparation of PVDF Composites

**Figure 1:**
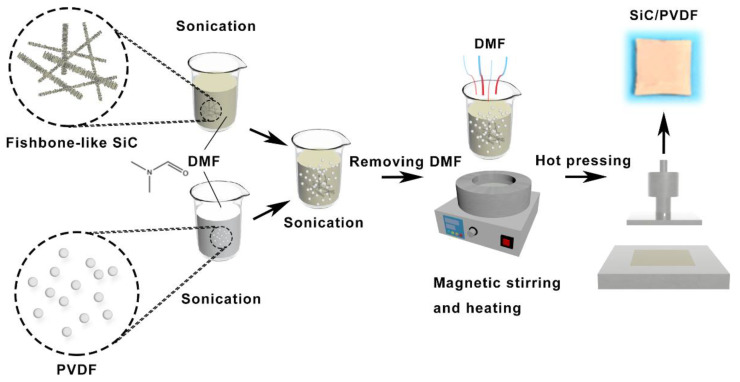
Preparation process of SiC/PVDF composites.

Figure 1 illustrates the preparation process of SiC/PVDF composites with different loadings of Fishbone-like SiC filler. Initially, SiC and raw PVDF powders were dispersed in DMF solutions by two beakers, respectively. After ultrasonic treatment of 5 min, the SiC/DMF and PVDF/DMF solutions were mixed into another beaker and ultrasonically blended for 20 min to obtain the mixing solution. In order to evaporate the DMF, the prepared solution was put into an oil bath pot at the temperature of 210 °C and magnetic stirring for 6 h. Finally, the SiC/PVDF composites were obtained by hot-pressing the remaining solute at 210 °C under 10 MPa for 30 min.

### 2.3. Characterization

The morphology of the Fishbone-like SiC filler, SiC/PVDF composites and pure PVDF was studied by a cold-field high-resolution scanning electron microscope (SEM, Regulus8230, HITACHI, Japan). The Raman spectrum of SiC powders was obtained by a Raman spectrometer (Raman, in Via Reflex, Renishaw, UK) using a laser with a wavelength of 532 nm. X-ray photoelectron spectroscopy (XPS, AXIS SUPRA, Kratos, UK) was performed with a Kratos axis ultra-DLD spectrometer, using aluminum Kα excitation radiation (hγ: 1253.6 eV). The thermal conductivity of the samples can be calculated by the formula $\lambda =\alpha \times C_{p}\times \rho$, where α, $C_{p}$, and ρ represent thermal diffusivity, specific heat capacity, and density, respectively. The thermal diffusivity (α) and specific heat capacity ($C_{p}$) were measured by a laser flash apparatus (LFA 467 HyperFlash, Netzsch, Germany). The density was obtained by the liquid displacement method. An infrared (IR) camera (Ti400, Fluke, USA) was used to record the infrared images of the obtained samples.

## 3. Results and Discussion

**Figure 2:**
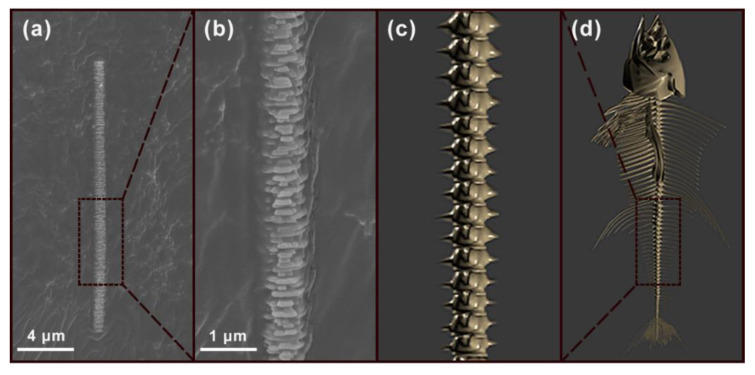
(**a**) and (**b**) SEM images of Fishbone-like SiC; (**c**) and (**d**) schematic diagrams of a fish skeleton.

Figure 2a,b show SEM images of individual special Fishbone-like SiC dispersed on conductive glue. Compared with the fishbone model in the comparison picture (Figure 2c,d), this SiC had an uneven diameter of about 600–800 nm, a length of about 16 μm, exhibiting jagged, rough topography. It is not difficult to imagine that the Fishbone-like SiC has a larger specific surface area than the straight and smooth SiC. However, according to reports in the literature, fillers with a high aspect ratio or specific surface area can form a more continuous thermal network in the polymer matrix so that the heat transfer efficiency between the thermally conductive filler and the polymer is greatly improved [35]. This is of great significance for the thermal conductivity improvement of composite materials.

**Figure 3:**
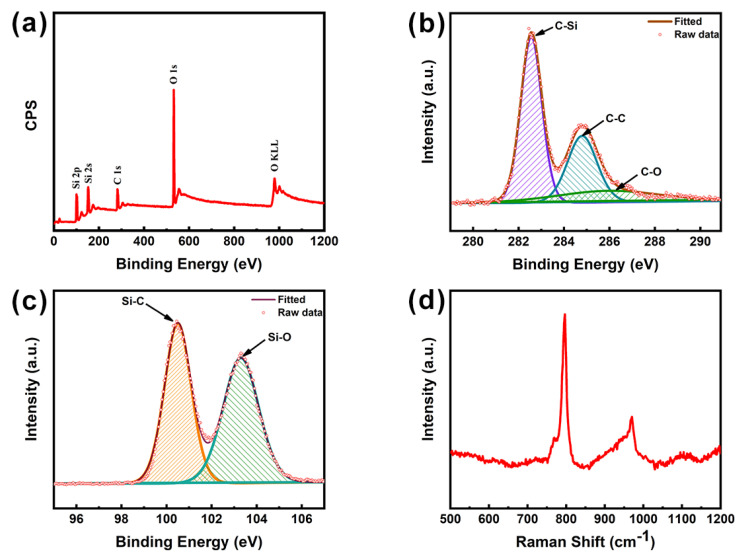
XPS spectra of SiC: (**a**) survey spectra, (**b**) C1S, and (**c**) Si2p; (**d**) Raman pattern of SiC.

As shown in Figure 3, XPS and Raman spectroscopy was used to further analyze the elemental component and bonding structure, respectively. In Figure 3a, the XPS spectrum proves that C, Si, and O were the only constituent elements of SiC. The high-resolution results of C1s and Si2p are shown in Figure 3b,c. The binding energy obtained in XPS analysis was charged to C1s based on 284.8 eV. The spectrum of C1s consisted of three peaks centered at 282.5, 284.8, and 286.1 eV, corresponding to C-Si, C-C, and C-O (Figure 3b). The spectrum of Si2p consisted of two peaks centered at 100.5 and 103.3 eV, corresponding to Si-C and Si-O, respectively (Figure 3c). In Figure 3d, the Raman spectrum shows that the two sharp peaks of 796 and 970 cm^−1^ corresponded to the absorption bands of SiC, which represent the horizontal and vertical Si-C bond vibrations, respectively. All the evidence proves the simultaneous existence of SiC and SiO_2_ [36].

**Figure 4:**
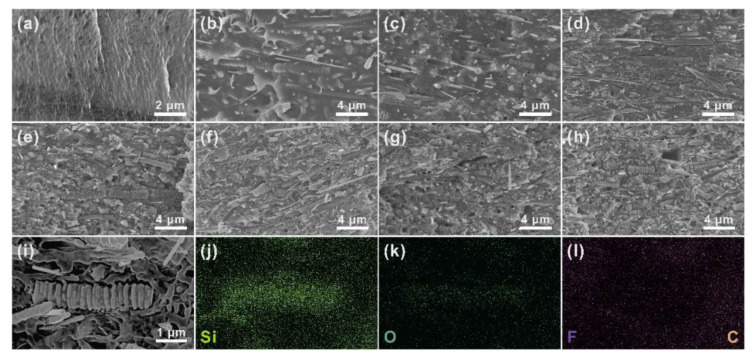
(**a**) SEM image of cross section of pure PVDF. Sectional SEM images of SiC/PVDF with (**b**) 10 wt%, (**c**) 20 wt%, (**d**) 30 wt%, (**e**) 40 wt%, (**f**) 50 wt%, (**g**) 60 wt%, and (**h**) 70 wt% filler content. (**i**) Enlarged SEM image of the 70 wt% SiC/PVDF and corresponding EDS mappings of (**j**) Si, (**k**) O, (**l**) F and C elements.

In order to further understand the relationship between the structure and performance of polymer composites, as shown in Figure 4, SEM was used to characterize the cross-sectional morphology of pure PVDF and PVDF composites with different filler content. Figure 4a shows the cross-sectional morphology of pure PVDF with strip-shaped cracks that are smooth and similar to rivers. As exhibited in Figure 4b–h, with the Fishbone-like filler content increasing, more and more SiC particles could be obviously exposed in the PVDF matrix and could gradually form cross-linked networks. Additionally, the sectional morphology of Figure 4g,h shows that there were a few pores that were generated by the hot-pressing process between the fillers and the PVDF matrix. The formation of pores was caused by the inferior fluidity of composites with high filler content. In addition, during the hot-pressing process, the high aspect ratio SiC added to the substrate was more manageable when forming a uniform orientation perpendicular to the hot-pressing direction [37,38,39,40,41], Figure 4j–l presents the energy dispersive spectroscopy (EDS) mapping spectrum in the region of Figure 4i, revealing the distribution of Si, O, F, and C elements. From the figure, we can clearly see that the Si elements were continuously mapped in a region and formed the outline of the SiC containing only the Si elements. At the same time, we observed that the mapping distribution of the O element was similar to that of the Si element. We can infer that the SiC fillers were surrounded by a layer of SiO_2_. Hwang et al. performed surface oxidation treatment on SiC after HF etching, enhancing the wettability between SiC particles and epoxy because there was a secondary interaction between the SiC particles and the epoxy [42]. The surface treatment of SiC particles enhanced the interface interaction between the particles and the epoxy, which reduced the interface thermal resistance and phonon scattering. The presence of a SiO_2_ shell enhanced the interface interaction between the filler and PVDF through the hydrogen bond function between -OH groups of SiO_2_ and the F atoms of PVDF (Figure S1), which reduced the interface thermal resistance and phonon scattering. Moreover, under the load of 70 wt% filler, the Si elements were connected to each other, proving that SiC was well connected in the PVDF matrix.

**Figure 5:**
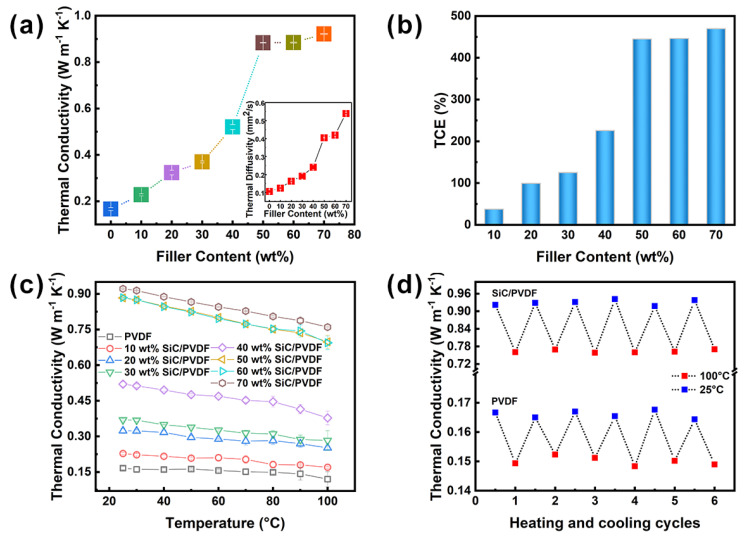
(**a**) Thermal conductivity, thermal diffusivity, and (**b**) thermal conductivity enhancement (TCE) of pure PVDF and SiC/PVDF composites with different filler content. Thermal conductivity of pure PVDF and SiC/PVDF composites with different filler content (**c**) as the temperature changes and (**d**) under multiple heating–cooling cycles.

Figure 5 demonstrates the enhancement of thermal transportation performance of polymer composites by adding Fishbone-like SiC fillers with different loadings. In Figure 5a, the thermal diffusivity and thermal conductivity of SiC/PVDF composites both increase with the increasing of filler content, which increased from 0.11 to 0.54 mm^2^/s and from 0.17 to 0.92 W m^−1^ K^−1^, respectively. Additionally, compared with the thermal diffusivity and thermal conductivity of samples with 0–30 wt% filler content, the thermal diffusivity and thermal conductivity of composites with more than 30 wt% filler content ascended quickly. The reason is that when the filling amount was less than one quantitative value, the formation of cross-linked networks was difficult because of the weak contact of fillers. When the fillers amount reached a certain value (percolation thresholds), the formation of thermal conductive networks benefited from the effective interaction between the fillers, providing convenience for phonon transmission [43,44,45]. As observed from the composites with 50 to 70 wt% filler content, the growth rate of thermal diffusivity and thermal conductivity slowed down, and a plateau appeared. The reason is that the thermally conductive networks had basically formed, and unceasingly, increasing the content of the fillers had little effect on the improvement of the thermal conductivity networks. Moreover, with the addition of a large number of fillers, the wettability relationship between the filler and the polymer was weakened, and a few pores appeared at the same time, referring to Figure 4f–h sectional SEM images. The thermal conductivity enhancement (TCE) rates of SiC/PVDF composites are summarized in Figure 5b. As the filler content increased, TCE could be calculated as follows:

(1) $$TCE=\left(\lambda_{1}-\lambda_{0}\right)/\lambda_{0}\times 100\%$$

where *λ*_0_ and *λ*_1_ are thermal conductivity of pure polymer and polymer composite, respectively. It was found that the changing trend of thermal conductivity enhancement (TCE) was consistent with the thermal conductivity change with the filler content increase, and the thermal conductivity of SiC/PVDF composite with the highest filler content was 470% higher than that of pure PVDF. Table S1 lists several PVDF composites with various thermal conductive fillers, where the Fishbone-like SiC/PVDF composite exhibited higher TC value compared with other works. Additionally, Figure 5c presents the thermal conductivity of pure PVDF and composites with different filler content depending on temperature changes. With a rise in temperature from 25 to 100 °C, the thermal conductivity of all samples exhibited a different decrement. The decrement rate of thermal conductivity of SiC/PVDF composite with 70 wt% filler content reached 17.5%, which was the lowest rate of all samples. The SiC/PVDF composite with 70 wt% filler content had the best heat dissipation ability among the eight samples. In addition, Figure 5d shows the stability of thermal conductivity undergoing 6 times of heating–cooling cycles. It can be seen that thermal conductivity of SiC/PVDF composite with 70 wt% fillers exhibited a slight change after the whole process of the heating–cooling cycle.

**Figure 6:**
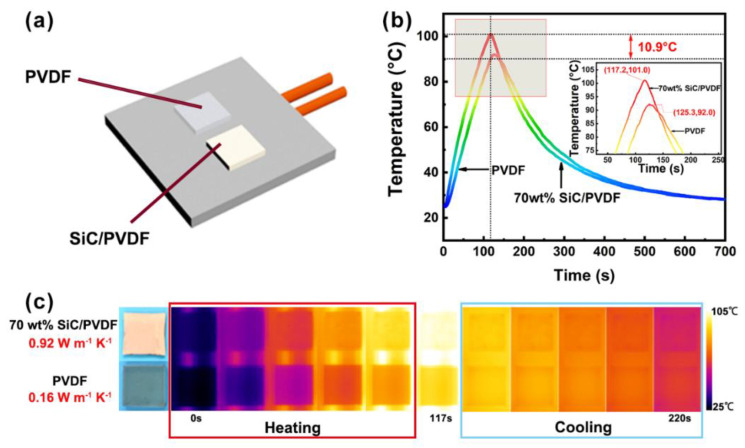
(**a**) Schematic diagram of heating device; (**b**) temperature–time curves, and (**c**) corresponding infrared images of 70 wt% SiC/PVDF composite and pure PVDF during and after heating.

In order to show the excellent thermal conduction performance of the SiC/PVDF composite visually, an infrared camera was utilized to measure and record the surface temperature of samples during and after heating. As presented in Figure 6a, pure PVDF and SiC/PVDF with 70 wt% filler content were located on a heating plate parallelly, and the two samples could be heated simultaneously after the silicon plate was linked with power. The recorded temperature–time curves and the corresponding IR images are shown in Figure 6b,c. After heating to 117 s, the temperature of the SiC/PVDF increased to 101 °C, which is 10.9 °C higher than that of pure PVDF. In addition, after the silicon plate stop being heated, the SiC/PVDF composite presented a faster temperature decrement compared with pure PVDF. Meanwhile, it can be found that the temperature of the pure PVDF after the plate stopped being heated continued to rise. This is because the thermal conductivity of pure PVDF is lower than that of the SiC/PVDF composite, and the temperature of pure PVDF at that time was still lower than that of the silicon plate. As exhibited in Figure 6c, the corresponding IR images show the same trend of temperature increasing and decreasing. In particular, the sample with a higher surface temperature showed a brighter color in the IR images. The experiments mentioned before testify that the SiC/PVDF composite containing Fishbone-like SiC possessed excellent heat conductive ability in the work situation.

## 4. Conclusions

In summary, the SiC/PVDF composites with different filler loading were obtained by hot-pressing. The rough surfaced SiC, which has high specific surface area, was similar to fish bone, which is conductive to the contact between the fillers. Due to the presence of SiO_2_ on the surface of Fishbone-like SiC, the interface interaction and the wettability between the filler and PVDF were enhanced. With the continuous increase in the filler content, the thermal conductivity of polymer composites was enhanced, which is attributed to the formation of a thermally conductive network in the polymer composite. The thermal conductivity of the PVDF composite with 70 wt% Fishbone-like SiC content reached 0.92 W m^−1^ K^−1^, which is 470% higher than pure PVDF. In addition, the infrared image further proves that its heat conduction capability is far better than pure PVDF. Thus, the Fishbone-like SiC provides new ideas for the application of polymer in the thermal management of electronic components.

## Data Availability

All data used and/or analyzed during the current study are shown within the study. If further data are required, it can be made available by the corresponding author upon reasonable request.

## Supplementary Materials

The following are available online at https://www.mdpi.com/article/10.3390/nano11112891/s1, Figure S1: The hydrogen bond function between -OH groups of SiO_2_ and the F atoms of PVDF enhances the interfacial interactions and compatibility between Fishbone-like SiC and PVDF, Table S1: Comparison of different thermal conductivity of PVDF composites.
