# Supplementary material for: Enhanced Thermal Conductivity of Polymer Composite by Adding Fishbone-like Silicon Carbide
Source: Nanomaterials (Basel). 2021 Oct 28;11(11):2891. doi: 10.3390/nano11112891 (PMC8620080; doi:10.3390/nano11112891)
Supplement: Supplementary file 1 [file nanomaterials-11-02891-s001.zip › nanomaterials-1411135-supplementary.pdf]

Supporting Information

Enhanced Thermal Conductivity of Polymer Composite by Adding Fishbone-like Silicon Carbide

Juncheng Xia[1,2#], Yue Qin[2#], Xianzhe Wei[2], Linhong Li[2,3], Maohua Li[2], Xiangdong Kong[2], Shaoyang Xiong[2], Tao Cai[2,3], Wen Dai[2,3], Cheng-Te Lin[2,3], Nan Jiang[2,3], Shuangquan Fang[1*], Jian Yi[2,3*], Jinhong Yu[2,3*]

[1]School of Mechanical Engineering, Yangzhou University, Yangzhou, Jiangsu 225009, China.

[2]Ningbo Institute of Materials Technology and Engineering, Chinese Academy of Sciences, Ningbo, Zhejiang 315201, China.

[3]Center of Materials Science and Optoelectronics Engineering, University of Chinese Academy of Sciences, Beijing 100049, China.

[#]These authors contributed to the work equally and should be regarded as co-first authors.

*Correspondence and requests for materials should be addressed to S. Fang (E-mail: sqfang@yzu.edu.cn), J. Yi (Email: yijian@nimte.ac.cn) or J. Yu (Email: yujinhong@nimte.ac.cn)

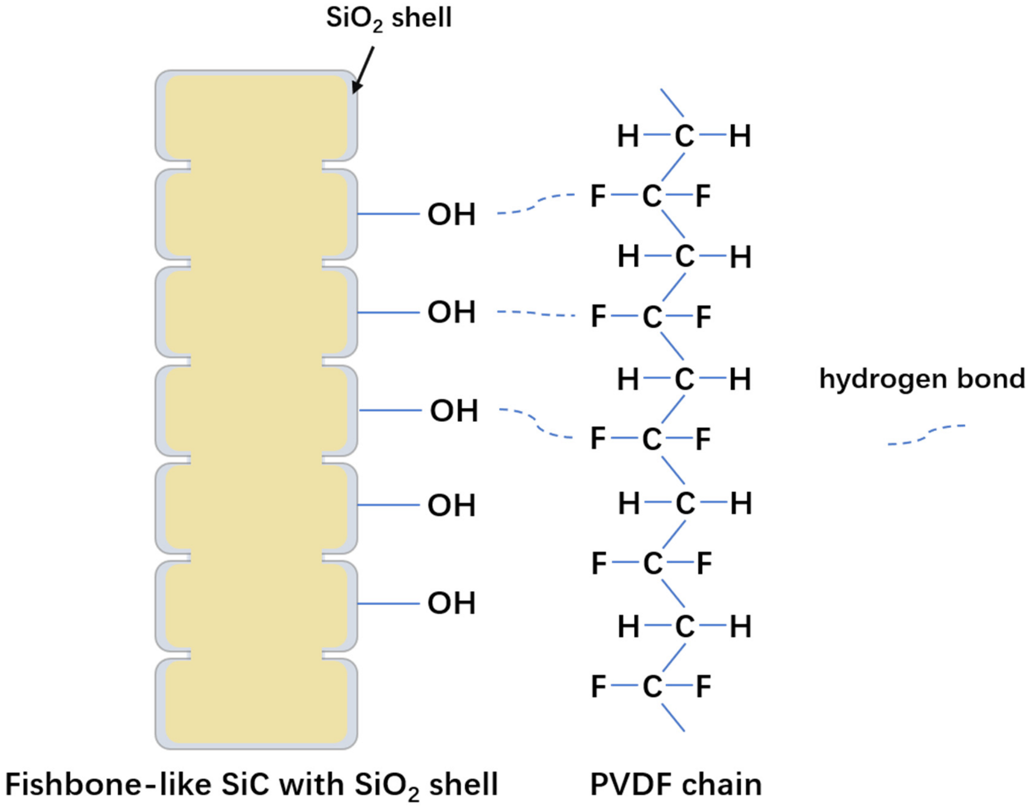

Figure S1 The hydrogen bond function between -OH groups of SiO_2 and the F atoms of PVDF enhances the interfacial interactions and compatibility between fishbone-like SiC and PVDF.

Table S1. Comparison of different thermal conductivity of PVDF composites.

Composites	Thermal conductivity (W m^{-1} K^{-1})	Reference
**This work**	**0.92**	
PVDF/BNNS (40 wt%)	0.82	[1]
PVDF/VmCNT (10 wt%)	0.51	[2]
PVDF/FGS@ND (45 wt%)	0.66	[3]
PVDF/PA/EG (3 vol%)	0.48	[4]
PVDF/Si@SiO_2@PS (50 wt%)	1.0	[5]
PVDF/f-SiC (13.8 vol%)	~0.35	[6]
PVDF/SiC@PDA@Ag (40 wt%)	0.43	[7]

Reference

1. Lei, Y.Z.; Liang, M.; Chen, Y.; Zhou, S.T.; Zou, H.W. Crystallization and thermal conductivity of poly (vinylidene fluoride)/boron nitride nanosheets composites. *Polym.-Plast. Tech. Mater.* **2020**, *59*, 1552-1561, doi:10.1080/25740881.2020.1757104.
2. Du, C.Y.; Li, M.; Cao, M.; Feng, S.C.; Guo, H.; Li, B.A. Enhanced thermal and mechanical properties of polyvinlydene fluoride composites with magnetic oriented carbon nanotube. *Carbon* **2018**, *126*, 197-207, doi:10.1016/j.carbon.2017.10.027.
3. Yu, J.H.; Qian, R.; Jiang, P.K. Enhanced thermal conductivity for PVDF composites with a hybrid functionalized graphene sheet-nanodiamond filler. *Fiber. Polym.* **2013**, *14*, 1317-1323, doi:10.1007/s12221-013-1317-7.
4. Tong, J.; Zhang, H.C.; Li, W.; Chen, H.C.; Wang, D.Y.; Hu, M.F.; Wang, Z.F. Simultaneously improving thermal conductivity and dielectric properties of poly(vinylidene fluoride)/expanded graphite via melt blending with polyamide 6. *J. Appl. Polym. Sci.* **2021**, *138*, 51354, doi:10.1002/app.51354.
5. Li, X.; Zhou, W.Y.; Cao, D.; Li, Y.; Li, T.; Chen, F.X.; Wu, H.J.; Wang, G.H.; Liu, X.R.; Cai, H.W.; et al. PVDF composites filled with core–shell fillers of Si@SiO_2, Si@SiO_2@PS: effects of multiple shells on dielectric properties and thermal conductivity. *J. Mater. Sci.-Mater. Electron.* **2021**, *32*, 23429–23444, doi:10.1007/s10854-021-06831-4.
6. Wang, B.; Yin, X.H.; Peng, D.; Lv, R.H.; Na, B.; Liu, H.S.; Gu, X.B.; Wu, W.; Zhou, J.L.; Zhang, Y. Achieving thermally conductive low loss PVDF-based dielectric composites via surface functionalization and orientation of SiC nanowires. *Express Polym. Lett.* **2020**, *14*, 2-11, doi:10.3144/expresspolymlett.2020.2.
7. Tong, Y.Z.; Zhao, W.J.; Wu, W.; Zhang, D.L.; He, G.J.; Yang, Z.T.; Cao, X.W. Realizing enhanced dielectric and mechanical performance of polyvinylidene fluoride/SiC nanocomposites through a bio-inspired interface design. *Adv. Compos. Hybrid Mater.* **2021**, doi:10.1007/s42114-021-00333-x.
